# Exercise Timing in Sport: Molecular and Physiological Mechanisms Linking Performance, Recovery, and Biological Cost

**DOI:** 10.3390/ijms27104415

**Published:** 2026-05-15

**Authors:** Dan Cristian Mănescu, Mirela Stoian, Mihaela Loredana Rădulescu, Corina Claudia Dinciu, Iulius Radulian Mărgărit, Marian Năstase, Cătălin Octavian Mănescu

**Affiliations:** 1 Department of Sport and Exercise Science, Bucharest University of Economic Studies, 010374 Bucharest, Romania; dan.manescu@defs.ase.ro (D.C.M.); mihaela.radulescu@defs.ase.ro (M.L.R.); catalin.manescu@defs.ase.ro (C.O.M.); 2 Department of AgriFood and Environmental Economics, Bucharest University of Economic Studies, 010374 Bucharest, Romania; mirela.stoian@eam.ase.ro; 3 Department of Management, Bucharest University of Economic Studies, 010374 Bucharest, Romania; marian.nastase@man.ase.ro

**Keywords:** chronotype, circadian rhythm, skeletal muscle clock, BMAL1, exercise timing, athletic performance, training load, heart rate variability, mitochondrial dynamics, redox biology, competition scheduling, ecological validity

## Abstract

Time of day is often treated as a scheduling constraint rather than a true programming variable, yet training time is not neutral and can modulate both performance expression and the biological cost of exercise. This structured narrative review synthesizes evidence on morning versus afternoon/evening training in athletes and physically active adults, integrating performance outcomes with internal load, autonomic recovery, sleep interaction, and downstream physiological stress. Acute evidence generally supports superior late-afternoon or early-evening performance, particularly for neuromuscular tasks, whereas chronic training studies do not indicate a universal optimal training time. Instead, adaptation appears to depend on chronotype, habitual training time, the wake-to-training interval, light exposure, nutritional timing, training–testing congruency, and competition-specific demands. Mechanistically, exercise timing likely interacts with BMAL1/CRY loops, AMPK-SIRT1-PGC-1alpha signaling, PGC-1alpha-driven mitochondrial remodeling, and redox-inflammatory pathways, although much of this evidence remains preclinical. Accordingly, this review advances a Performance–Biological Cost (PBC) framework in which training time is judged by the balance between output, recovery opportunity, and residual molecular cost rather than by acute performance alone. By integrating athlete-relevant human evidence with mechanistic insights and translating them into explicit decision tools and testable predictions, this review positions exercise timing as a context-dependent lever within periodized training design.

## 1. Introduction

Training time is not a neutral scheduling variable, but a context-dependent modulator of both performance output and biological cost. Circadian rhythms influence body temperature, autonomic tone, the endocrine background, substrate availability, and several dimensions of neuromuscular readiness, while skeletal muscle itself contains an intrinsic molecular clock. Exercise timing, therefore, alters both the physiological state upon which a session is imposed and the signaling environment through which that session is translated into adaptation [1,2,3,4,5].

Acute syntheses consistently report better late-afternoon or early-evening performance for maximal strength, power, sprint, repeated-sprint, and many endurance outcomes, although the average advantage is outcome- and protocol-dependent rather than universal [6,7,8,9]. Pooled effects appear to range from small for some endurance syntheses (effect size about 0.23) to moderate for time-to-exhaustion or total work outcomes (Hedges’ g about 0.65), and are larger for Wingate power and jump height (effect sizes about 0.73 and 0.79, respectively) [6,7,8]. The applied question is therefore not simply whether afternoon exceeds morning, but whether the same session produces the same combination of output, internal load, recovery opportunity, and downstream biological cost at different circadian phases.

This distinction matters because training quality must be judged across the microcycle rather than within a single session. A time slot that modestly improves immediate output may still be suboptimal if it compresses sleep, delays autonomic recovery, or increases residual fatigue before the next key practice. Conversely, strategically protected morning work may be appropriate when athletes must rehearse for early competition, accommodate unavoidable logistics, or preserve evening recovery [10,11,12,13,14]. The ecological cost of training therefore extends beyond same-session performance and must be interpreted across the wider 24 h recovery architecture.

The familiar morning-versus-evening comparison is only a proxy for a broader chronoecology. In practice, athletes perform within a phase relationship between internal circadian timing and external demands, shaped by light exposure, meal timing, ambient temperature, and proximity to sleep. This helps explain why apparently conflicting findings can coexist, and why the most appropriate training slot often depends on chronotype, habitual training time, the wake-to-training interval, and competition-specific demands rather than on clock time alone [15,16,17,18].

Within this context, the present review integrates circadian regulation with redox homeostasis and metabolic signaling pathways relevant to exercise adaptation [19,20,21]. It advances a Performance–Biological Cost (PBC) model in which training time is judged by the balance between output, recovery opportunity, and residual molecular cost rather than by acute performance alone. Rather than searching for one universally optimal hour, this perspective treats exercise timing as a context-dependent programming variable whose practical value depends on how it shapes the ratio between performance output and biological cost across the wider recovery architecture.

### Research Questions and Aims

To address these gaps, the present structured narrative review is guided by the following key questions:

**Q1.** Does the time of day consistently influence acute exercise performance across different physical capacities?
**Q2.** To what extent does exercise timing modify internal load, perceived effort, and recovery processes, including sleep interaction?
**Q3.** What is the current evidence supporting circadian modulation of molecular pathways involved in exercise adaptation, including metabolic and redox signaling?
**Q4.** How do chronotype, habitual training time, and contextual factors (e.g., wake-to-training interval, light exposure, and nutrition) shape observed phase-dependent effects?
**Q5.** Can these multi-level effects be integrated into a coherent framework that explains how training time influences both performance and its associated biological cost?

By addressing these questions, the review aims to integrate athlete-relevant human evidence with mechanistic insights and to clarify how exercise timing may influence both performance expression and the biological cost of training.

## 2. Methodological and Conceptual Framework

### 2.1. Methodological Approach

This article is a structured narrative review designed to synthesize athlete-relevant human performance evidence together with mechanistic literature that can inform training-time decisions in athletes and physically active adults. Literature was searched iteratively in PubMed and Scopus from database inception to February 2026 using combinations of the terms *time of day*, *circadian*, *chronotype*, *exercise*, *training*, *performance*, *strength*, *power*, *sprint*, *endurance*, *heart rate variability*, *sleep*, *skeletal muscle clock*, *mitochondria*, *redox*, *inflammation*, *light exposure*, *nutrition*, and *jet lag*. Search strings were adapted iteratively across databases and, where appropriate, limited to human studies and English-language publications to maximize translational relevance. Reference lists of key reviews, meta-analyses, and eligible primary studies were also screened to identify seminal papers and recent updates.

The evidence base was organized around predefined translational themes corresponding to the research questions and to the main practical decision points faced in sport: acute performance output; internal load and perceived exertion; autonomic recovery, heart rate variability, and sleep interaction; chronotype, habitual training time, the wake-to-training interval, and training–testing congruency; co-zeitgebers such as light, meals, and caffeine; travel-related circadian misalignment; and molecular mechanisms linking the skeletal muscle clock with metabolic, mitochondrial, endocrine, and redox-inflammatory signaling. For each study, emphasis was placed on the study design, participant profile, exercise modality, timing comparison, contextual modifiers, and principal outcomes so that findings could be interpreted across levels of analysis rather than as isolated results.

Priority was given to meta-analyses, systematic reviews, randomized or crossover human trials, athlete field studies, and mechanistic human data. When multiple levels of evidence were available, athlete-based human studies were weighted most heavily, followed by mechanistic human studies, whereas preclinical studies were retained selectively to clarify biologically plausible pathways that remain difficult to isolate directly in sport settings. Studies in clinical or metabolically altered populations were included when they offered mechanistic insight into clock-sensitive pathways, metabolomic responses, or exercise-timing effects that remain difficult to characterize in trained athletes, but such evidence was interpreted as supportive rather than definitive for athletic prescription.

Study selection was guided by explicit relevance criteria rather than by formal systematic review procedures. Preference was given to studies that: (1) examined exercise timing in relation to performance, internal load, recovery, sleep, or molecular responses; (2) reported sufficient contextual detail to permit interpretation of phase-dependent effects, such as chronotype, habitual training time, wake time or time since waking, feeding status, or proximity to sleep; and (3) offered direct or translational relevance to sport, training design, or structured physical activity. Studies with insufficient contextual description were interpreted cautiously and were not allowed to drive applied conclusions. Ecological validity was considered throughout, particularly where laboratory findings required translation to real training calendars, competition schedules, travel demands, or congested microcycles. In cases of conflicting findings, greater weight was assigned to studies with higher ecological validity, athlete populations, and more complete contextual reporting, rather than to isolated statistically significant results.

Because the purpose of the review was integrative explanation rather than exhaustive cataloging or quantitative pooling, it was not conducted under a preregistered protocol and does not include a PRISMA flow diagram or formal risk-of-bias scoring. Conclusions are therefore framed according to the direction, consistency, ecological validity, and translational relevance of the available evidence rather than by numerical vote counting, with explicit separation between stronger human evidence and more provisional mechanistic inference. Evidence synthesis followed a thematic convergence approach, integrating findings across performance, recovery, and mechanistic domains to identify consistent patterns rather than relying on single-study outcomes. Particular caution was applied where the literature was male-dominated, relied on non-athlete cohorts, or inferred athlete-relevant mechanisms primarily from animal and cellular models.

Inclusion criteria included: (1) human or translational relevance; (2) reporting of performance, recovery, or molecular outcomes; and (3) sufficient temporal and contextual detail to enable phase-dependent interpretation. Studies lacking such contextual information were excluded or interpreted with caution and were not permitted to drive applied conclusions.

To increase transparency despite the non-systematic design, the evidence was mapped into predefined tiers according to source type, population, ecological proximity to sport, and inferential role. Ecological validity was operationalized by five criteria: (1) athlete or trained-participant relevance; (2) use of sport-specific or field-compatible tasks; (3) reporting of wake time, habitual training time, feeding, caffeine, light exposure, or the session-to-sleep interval; (4) connection to real training, competition, travel, or microcycle constraints; and (5) inclusion of recovery or next-day outcomes when the biological cost of a session was being inferred. Studies satisfying fewer criteria were retained only as supportive or mechanistic evidence and were not allowed to drive practical recommendations.

Because formal meta-analysis was not the purpose of this review, the certainty of key statements was summarized using a pragmatic evidence–confidence map rather than a formal GRADE procedure. Confidence levels were assigned from the convergence of study design, population relevance, consistency, ecological validity, and the directness of the outcomes: high confidence indicates repeated human syntheses or consistent athlete-relevant evidence; moderate confidence indicates consistent but heterogeneous human evidence; low confidence indicates limited human data, indirect populations, or a strong dependence on contextual modifiers; and very low confidence indicates primarily preclinical or hypothesis-generating evidence.

The evidence architecture and representative characteristics of the literature included in the structured narrative synthesis are summarized in Table 1.

To improve interpretability across the different evidence domains, the main chrono-exercise findings and their associated confidence levels are synthesized in Table 2.

### 2.2. Conceptual Contribution

Several prior reviews have addressed circadian variation in sport performance, chronotype, time-specific training, the skeletal muscle clock, or the broader interaction between exercise timing and metabolic health [3,4,5,6,16,17,18,19,20,55,56,57]. The present review extends that literature in three ways. First, it formalizes a PBC model that evaluates exercise timing through an output-to-biological-cost lens rather than through acute performance alone, explicitly integrating internal load, autonomic recovery, sleep interaction, and residual molecular stress. Second, it links athlete-focused human evidence to the molecular-clock, mitochondrial, endocrine, and redox-inflammatory literature while distinguishing between stronger human data and more provisional mechanistic inference. Third, it translates this synthesis into an applied decision matrix, integrative figures, and testable predictions intended to improve both practice and future chrono-exercise study design.

**Figure 1:**
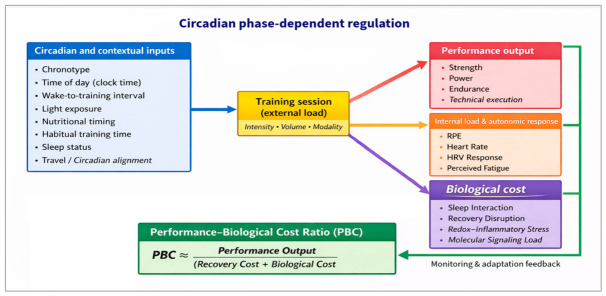
Performance–Biological Cost (PBC) framework for chrono-informed training design. Training outcomes emerge from the interaction between circadian and contextual inputs and the externally prescribed training load. A single session generates parallel responses across performance output, internal load, and biological cost. The PBC ratio conceptualizes training-time effectiveness as the balance between performance output and its associated recovery and molecular cost, embedded within a feedback loop driven by monitoring variables. The entire system operates within a circadian phase-dependent context [3,4,5,6,16,17,18,19,20,21,55,56,57].

Figure 1 illustrates the Performance–Biological Cost (PBC) framework, which formalizes training-time effectiveness as the balance between performance output and its associated biological cost within a circadian-dependent system.

Despite the growing body of literature, a critical limitation remains the relative scarcity of athlete-specific molecular evidence directly linking exercise timing to adaptive signaling pathways. Much of the current mechanistic understanding is derived from preclinical models or clinical populations, which, although biologically informative, may not fully capture the physiological complexity, training load, and ecological constraints characteristic of athletic environments. As a result, the PBC model should be read as an integrative and explicitly testable framework rather than as a claim that one clock hour can already be prescribed universally on molecular grounds.

## 3. Circadian Architecture Relevant to Training Timing

### 3.1. Biological Rhythms and Acute Exercise Capacity

The circadian system coordinates daily oscillations in physiology through a central pacemaker in the suprachiasmatic nucleus (SCN) and a network of peripheral clocks distributed across tissues, including skeletal muscle and metabolic organs [2,5,21]. For exercise, this matters because variables with clear daily rhythms—core temperature, alertness, sympathetic activity, cortisol, and melatonin—also influence readiness to perform. The athlete therefore enters each session with a circadian state that can facilitate or constrain the imposed load.

One of the most robust practical observations is that body temperature usually rises from the morning toward the late afternoon. Higher temperature is associated with faster nerve conduction, lower passive stiffness, improved muscle contractile properties, and more favorable enzyme kinetics, all of which can contribute to better high-intensity performance. Importantly, a thorough warm-up can reduce part of the morning disadvantage, but it does not necessarily eliminate it fully, particularly for explosive tasks or when the athlete is sleep deprived [1,58,59,60].

Endocrine rhythms add another layer of complexity. Cortisol is generally higher in the morning, whereas the broader late-day environment is characterized by different combinations of autonomic tone, perceived alertness, and metabolic readiness. These patterns should not be reduced to simplistic anabolic-versus-catabolic narratives; rather, they signal that the same external load may be processed differently depending on when it is applied [2,5,61].

### 3.2. Chronotype, Habitual Training Time, and Peripheral Clocks

Chronotype describes the individual preference for earlier or later sleep–wake timing and is one of the main reasons why the same clock hour does not feel biologically identical across athletes [4]. Morning-oriented individuals often tolerate early sessions better, whereas evening-oriented individuals may experience larger decrements in alertness, mood, and performance when required to train early. Chronotype is therefore not a lifestyle curiosity; it is an applied variable with direct implications for readiness and recovery.

Habitual training time also matters. Repeated exposure to training at a similar hour appears able to attenuate some time-of-day differences and improve performance at the trained time, probably through a combination of behavioral adaptation, anticipatory regulation, and repeated interaction with local peripheral clocks. In practice, this means that the athlete who always trains in the morning may express less of a morning penalty than a comparable athlete whose usual schedule is evening-based [3,10,51,52,53].

This adaptation is crucial for sport settings because training and competition are rarely scheduled for ideal biological times. School timetables, team logistics, travel, and broadcasting schedules create mismatches between chronotype and demanded performance windows. For that reason, research and practice should distinguish among at least three layers: the athlete’s inherent chronotype, the biological effects of a given clock hour, and the acclimation produced by repeated time-specific training [22,23].

A further limitation is that most sport studies define chronotype through questionnaires rather than through objective circadian phase markers. Morningness–eveningness questionnaires are useful and scalable, but they are not equivalent to dim light melatonin onset (DLMO), continuous temperature rhythm, actigraphy-derived sleep timing, or other physiological indices of phase. This distinction is especially relevant in athletes, whose sleep–wake patterns may be constrained by training, school, travel, or competition rather than freely expressing biological preference [42,76,77,78]. Consequently, chronotype should be interpreted as a practical screening variable, while objective phase assessment should be considered when high-stakes timing decisions, travel, or repeated early starts are involved.

### 3.3. Molecular Circadian Regulation of Skeletal Muscle and Exercise Signaling

Skeletal muscle contains an intrinsic molecular clock built around transcription–translation feedback loops. In the positive limb, BMAL1 and CLOCK bind E-box elements and promote rhythmic transcription of PER, CRY, REV-ERB, and ROR targets, whereas PER/CRY complexes repress BMAL1:CLOCK activity. This oscillator is not a decorative molecular feature. In muscle it regulates genes involved in glucose handling, substrate selection, myogenesis, contractile function, protein turnover, and mitochondrial homeostasis [19,20,21,61].

Genetic and tissue-specific models indicate that the muscle clock is functionally relevant for performance and metabolic health. Loss of BMAL1 or CLOCK signaling in muscle reduces specific force, disrupts sarcomeric organization, lowers mitochondrial volume and respiration, and impairs insulin-stimulated glucose uptake, partly through effects on GLUT4, TBC1D1, pyruvate dehydrogenase control, and SIRT1-linked pathways [62,63,64,65,66,67]. These data provide a mechanistic basis for why identical exercise can be biologically processed differently at different circadian phases.

Several exercise-responsive signaling hubs likely intersect directly with the clock, especially in experimental models and limited human work. AMPK can modify clock phase through effects on CRY stability, SIRT1 couples redox/energetic state to deacetylation of clock components, and PGC-1alpha co-regulates oxidative metabolism together with BMAL1:CLOCK and ROR-mediated transcription [19,20,61,65,68,69]. In practical terms, exercise timing is not merely superimposed on physiology; it likely interacts with the cellular machinery that interprets exercise.

Exercise is also a zeitgeber for peripheral tissues. Scheduled exercise can phase-shift skeletal muscle clocks even when the SCN is unchanged, and repeated training at a given hour can modify clock gene expression around that trained window [26,54,64,70,71,72,73]. This helps explain why habitual training time and training–testing congruency alter performance and adaptation.

### 3.4. Additional Zeitgebers, Wake-to-Training Interval, and Circadian Ecology of Training

Clock time alone is an incomplete descriptor of training biology. Time since waking, prior light history, meal timing, caffeine use, and ambient temperature can each shift readiness or alter the apparent magnitude of a phase effect. Thus, two sessions scheduled at the same nominal hour are not necessarily biologically equivalent when one occurs shortly after waking in dim light and the other follows a longer wake-to-train interval with bright light exposure, breakfast, and a deliberate warm-up [17,74,75,76,77].

Light and feeding deserve particular attention because they are potent zeitgebers for central and peripheral oscillators. Bright light exposure, melatonin suppression, meal timing, and the distribution of carbohydrate and protein across the day can alter circadian phase or create SCN–periphery mismatch, while mistimed light at night or late eating can disturb hormonal and metabolic rhythms [55,78,79,80,81,82]. For athletes, this means that part of the so-called morning penalty may sometimes reflect coexisting sleep inertia, incomplete fueling, low energy availability, or inappropriate light history rather than an immutable property of morning exercise [83]. Chrono-nutrition is therefore not an accessory concept but part of the biological context in which timed exercise is decoded.

Accordingly, training-time research should report wake time, time since waking, pre-exercise light exposure, and breakfast or caffeine timing whenever feasible. Practitioners can use these same variables as programming levers: protect the wake-to-train interval when possible, consider strategically timed light and carbohydrate intake, and avoid creating circadian confusion through repeated late-night light exposure followed by early-morning high-intensity work [17,75,78,79,80]. Objective phase markers may also add value because self-reported chronotype does not always capture internal circadian alignment in field settings, particularly in elite athletes exposed to travel, shift-like schedules, or irregular competition calendars [42].

Several apparently circadian effects may therefore be exaggerated or attenuated by modifiable pre-session conditions. Caffeine should be standardized by dose, timing, and habitual use; hydration should be controlled because plasma volume and cardiovascular strain can alter both performance and HRV; thermal state should be reported because passive heating or a stronger warm-up can reduce part of the morning disadvantage; and late light exposure, screen use, or delayed meals should be recorded because they may shift circadian phase or disturb sleep. In applied terms, these factors are not nuisances only; they are controllable levers that can lower the biological cost of a necessary early or late session [17,55,58,59,60,78,79,80,81,82,83].

## 4. Time-of-Day Effects on Performance Output

### 4.1. Neuromuscular and Team-Sport Performance

Meta-analytic and systematic-review evidence most consistently supports small but recurrent late-afternoon or early-evening advantages for tasks that depend heavily on neuromuscular expression, including maximal strength, jump performance, short sprinting, and repeated-sprint ability [6,8,9,24]. In the broader short-duration literature, peak performance is most often reported between about 16:00 and 20:00 h, with typical amplitudes in dynamic tasks ranging roughly from 1.7% to 17.5% rather than representing a universal all-day shift [8]. In team-sport athletes, even modest average gains can matter because sprint outcomes, duels, change-of-direction quality, and repeatability of high-intensity actions are often decided by narrow margins.

The mechanisms are probably multifactorial. Temperature-related improvements in muscle function interact with neural readiness, arousal state, perceived effort, and mechanical factors such as compliance and stiffness. Increasingly, mechanistic work also suggests that time-specific expression of clock-controlled metabolic and contractile pathways shapes how force and fatigue are generated, not only how warm or alert the athlete feels [21,62,70,71,72]. No single mechanism explains all studies, and not all athletes respond identically, but the overall pattern is sufficiently recurrent to justify considering late afternoon or early evening as a favorable window for high-expression speed–power work when schedules allow [1,2,60].

Recent sport-specific work supports this interpretation. In elite soccer players, morning and evening training produced different effects on both performance and well-being, with evening sessions associated with a better mood profile and superior small-sided game responses [43]. For applied sport science, this is important because technical–tactical quality is often inseparable from neuromuscular freshness and the willingness to tolerate high intensity.

### 4.2. Endurance Performance

Endurance outcomes are more heterogeneous than explosive tasks, but recent syntheses still tend to show a late-day advantage in many acute settings [6,7]. The effect is usually smaller and more context-sensitive than for strength or sprint performance, with a meaningful dependence on test duration, intensity domain, pacing strategy, thermal conditions, feeding status, and prior sleep.

This heterogeneity has an important practical implication. A seemingly circadian effect may partly reflect modifiable contextual variables. Morning testing is often accompanied by earlier waking, reduced sleep, lower body temperature, incomplete pre-exercise fueling, or a fasted state. Therefore, coaches should be careful not to interpret every morning decrement as a fixed biological destiny. Some of the observed disadvantage may be reduced by better sleep protection, earlier meal planning, and a more deliberate warm-up.

Even so, the applied goal is not to erase every diurnal difference. For sports contested in the morning, training must sometimes prepare athletes to express endurance performance under morning conditions. In that context, time-specific exposure becomes part of competition specificity rather than an error in programming.

### 4.3. Acute Responses Versus Chronic Adaptation

A crucial distinction is that the hour of best acute performance is not necessarily the hour of best long-term adaptation. Several time-specific training studies suggest that repeated training at a given hour improves subsequent performance measured at that same hour, indicating a training–testing congruency effect rather than universal superiority of one time across all outcomes [10,51,52,53].

This point is reinforced by recent work showing limited evidence for large differential chronic adaptation when resistance training is performed in the morning versus the evening under controlled conditions [27]. At the same time, recent randomized trials and exercise training studies indicate that morning and evening programs can diverge in glycemic control, body mass change, sleep, cardiometabolic markers, or hypertrophic/strength outcomes depending on phenotype, exercise mode, and the outcome selected [28,29,30,31,32,33,34]. In other words, the clock time that maximizes same-day output may not always determine the clock time that produces the greatest accumulated benefit across weeks.

For coaches, the implication is simple but profound: the best time to express performance today and the best time to place a session within a microcycle are related questions, but they are not identical. Timing decisions become stronger when they are judged against the total ecological cost of the session rather than by acute output alone.

### 4.4. Molecular Evidence Linking Clock Time to Acute Output and Adaptation

Mechanistic omics studies sharpen this picture, but they should be interpreted through a hierarchy of translation. Rodent and cellular work provides valuable causal evidence that PER1/2, HIF-1alpha-linked carbohydrate use, BMAL1/CLOCK regulation, and phase-specific transcriptomic programs can alter exercise capacity and tissue remodeling [62,63,64,65,66,67,68,69,70,71,72]. However, these models do not by themselves justify athlete prescriptions. In the present review, preclinical findings are therefore retained as mechanistic scaffolding only, whereas applied recommendations are anchored primarily in human syntheses, athlete field data, and the small number of human mechanistic studies that report exercise time effects on metabolomic, proteomic, clock gene, or circadian-phase outcomes [6,7,8,9,26,42,43,44,54].

The clearest translational priority is to separate three levels of inference: (1) athlete–human evidence, which supports timing decisions for performance and recovery; (2) clinical or metabolically altered human evidence, which shows that exercise time can modify multi-tissue metabolomic or skeletal muscle clock responses but may not generalize directly to trained athletes; and (3) preclinical evidence, which identifies candidate pathways for future tests. Under this hierarchy, studies such as Savikj et al. [26] and Harmsen et al. [54] are more directly relevant to human chrono-exercise biology than mouse-only experiments, but they still remain indirect for elite sport prescription.

Accordingly, the molecular contribution of the present review is deliberately hypothesis-generating. The most testable proposition is that clock time modifies the output-to-cost ratio by altering the basal metabolic, redox, and autonomic state on which exercise is imposed. Future athlete studies should test whether time-specific training changes local clock gene expression, substrate signatures, mitochondrial remodeling, and next-day recovery in parallel with sport-specific output, rather than inferring prescription from molecular plausibility alone [26,54,97,100,108,109].

### 4.5. Competition Scheduling, Ecological Validity, and Training–Testing Congruency

A recurrent problem in chrono-exercise interpretation is that laboratory peak time is too quickly equated with competition advantage. Yet real competition includes psychological activation, tactical context, travel, warm-up resources, environmental stress, and the degree of alignment between local time and the athlete’s internal phase. Olympic-level field data confirm that time of day can meaningfully influence competitive outcomes, but they also show that ecological context determines whether the biological signal is amplified, dampened, or masked [44].

The concept of training–testing congruency is therefore central. Athletes improve not only because they train, but because they repeatedly rehearse the state in which performance must later be expressed. Chronotype, habitual training time, time since waking, and objective circadian phase each change the size and direction of diurnal performance effects [42,75,84,85,86,87]. Thus, a program made entirely of late-day high-quality work may build impressive afternoon output while leaving the athlete underprepared for morning heats, qualifiers, or travel-compressed starts.

This issue is especially relevant in team and professional sport, where circadian misalignment between local time, habitual schedule, and competition time can erode decision quality and physical output. Evidence from elite sport settings suggests that performance decrements are not merely theoretical when athletes are asked to operate at misaligned circadian phases [45,46,47,48].

Competition scheduling should therefore be treated as both an applied target and a potential confounder. Intensive calendars create repeated perturbations in sleep opportunity, travel exposure, residual muscle damage, emotional activation, nutritional routines, and taper structure. These factors can make a morning session appear biologically costly because it follows a late match, or make an evening session appear favorable because it coincides with a better-rested phase of the calendar. Future studies and applied monitoring reports should document at minimum, the start time of recent and upcoming competitions, days since the last event, days to the next event, match minutes or race load, travel direction, and time-zone history over the previous 7–14 days [44,45,46,47,48,78,79,80].

Table 3 and Table 4 condense the main athlete-level modifiers, evidence domains, and practical implications.

## 5. Internal Load, Perceived Exertion, and Mood

External load describes what was prescribed; internal load reflects what it cost the athlete to complete it. Heart rate, blood lactate, session rating of perceived exertion, mood, and perceived recovery all belong to this internal side of the equation. Timing matters because the same external session can be experienced through different physiological and psychological states depending on the time of day [5,22,43].

From an applied standpoint, this point may be even more important than the modest average differences in acute output reported by meta-analyses. If a session performed at one hour produces similar total work but with lower perceived strain, better mood, or less disruption to subsequent recovery, that timing may be preferable over weeks of training. This is particularly relevant in team sports, where the quality of decision-making, communication, and technical execution can deteriorate before a large drop is a visible in external metrics.

The study by Selmi and colleagues illustrates this logic well: evening training in elite soccer players was associated not only with better performance indicators but also with more favorable well-being responses [43]. Such findings encourage coaches to interpret timing through a broader lens. A successful session is not merely a session that hits the planned wattage or speed; it is also a session that fits the athlete’s readiness profile and leaves an acceptable residue for the next training demand.

Morning training is not therefore a mistake. It can be strategically important for acclimation to morning competition, for facility availability, or for the organization of double-session days. The lesson is simply that when morning training is necessary, it should be supported with better sleep opportunity, deliberate thermal preparation, and careful nutrition so that internal load is not inflated unnecessarily.

In skill-dominant and tactically dense environments, timing can also change the quality of learning that occurs within the session. Sleepiness, chronotype mismatch, and inadequate readiness may raise perceived effort before a major drop becomes visible in gross external metrics. For that reason, coaches should track not only the physical load completed, but also the technical and cognitive quality that the chosen time slot is able to support [43,44,84].

## 6. Autonomic Recovery, Heart Rate Variability, and Sleep

### 6.1. Session-to-Sleep Interval, Morning Sleep Compression, and Recovery Quality

Heart rate variability (HRV) is widely used as a non-invasive window into autonomic state and recovery, yet its relationship with time of day and chronotype remains underexplored relative to performance outcomes [22,23]. That gap matters because recent athlete- and sleep-focused reviews now make clear that performance cannot be separated from the broader sleep–circadian context in which training is placed [25,88,89,90,91,92]. Training decisions should therefore be based not only on what happens during exercise, but also on how quickly the athlete returns toward physiological stability afterward.

HRV should also be interpreted with methodological restraint. It is sensitive to hydration status, heat exposure, emotional stress, illness, alcohol or caffeine use, menstrual symptoms, sleep debt, measurement posture, and residual sympathetic activation after late intense exercise. A lower morning HRV after an evening session may therefore indicate incomplete recovery, but it may also reflect transient autonomic carryover from a demanding stimulus rather than maladaptation. For PBC use, HRV is best interpreted as a trend and in combination with sleep, session RPE, mood, soreness, and next-day performance rather than as a standalone proxy for biological cost [22,23,35,36].

Available evidence suggests that chronotype and exercise timing can influence autonomic recovery. Acute work in athletes has shown that high-intensity exercise may disturb cardiac autonomic function differently according to chronotype, and older research indicates that post-exercise parasympathetic reactivation also varies across times of day and chronotype profiles [35,36]. These findings are not yet strong enough to support rigid rules, but they clearly argue against assuming that all athletes absorb an early or late session in the same way.

Timing must also be considered in relation to the sleep window. Early-morning training can reduce sleep duration severely in athletes who need to travel, wake early, or manage two-a-day schedules [12,14]. At the opposite end of the day, the evening-exercise literature no longer supports a simplistic prohibition: high-intensity or late sessions do not uniformly impair sleep, but their effects depend on dose, finish time, time-to-bed, chronotype, prior sleep debt, and the athlete’s broader sleep behaviors [11,37,38,39,40,41,88]. As a conservative programming anchor, vigorous HIIT, repeated-sprint, or heavy resistance sessions should usually finish at least about 2–3 h before attempted sleep when sleep protection is a priority, whereas low-intensity recovery or technical work may be tolerated closer to bedtime in well-adapted athletes. Conversely, when quality work must occur in the morning, a wake-to-training interval of roughly 90–180 min is preferable for high-output sessions when logistics permit, with shorter intervals reserved mainly for low-intensity or competition-specific rehearsal. These are decision ranges, not universal thresholds.

In athletes, this issue is amplified by travel, competition, sex-specific sleep vulnerability, and the quality of sleep behaviors around training. Recent systematic reviews and cohort studies show that sleep interventions can improve performance in some contexts, whereas disrupted sleep and circadian rhythms are associated with poorer recovery and mental health outcomes, especially in female athlete cohorts [49,50,90,91,92]. Chrono-aware session placement should therefore be interpreted as part of sleep management, not as an isolated scheduling choice.

This creates a practical tension. The late afternoon or early evening may favor performance expression, but a poorly timed evening session can compromise recovery if it extends into the pre-sleep period. For some athletes, a late-afternoon finish offers the best compromise between high output and preserved sleep; for others, especially pronounced evening types, early-competition athletes, or those under dense schedules, a blend of strategic morning exposure, protected sleep opportunity, and controlled pre-session fueling/light may produce a better PBC ratio [11,12,13,14,37,38,39,40,41,88].

### 6.2. Travel, Jet Lag, and Circadian Misalignment

Travel and time-zone shifts transform a simple clock-time question into a phase misalignment problem. Eastward travel, dense schedules, night arrivals, and altered meal timing can degrade sleep, autonomic stability, mood, and skill execution. Consensus guidance therefore emphasizes staged phase shifts, light management, melatonin where appropriate, and deliberate timing of meals and training after arrival [46,47,48,78,79,80]. Recent field work further suggests that objective melatonin timing can reveal circadian misalignment beyond self-reported chronotype in elite female footballers, highlighting the value of phase-aware monitoring when calendars become biologically hostile [42].

**Figure 2:**
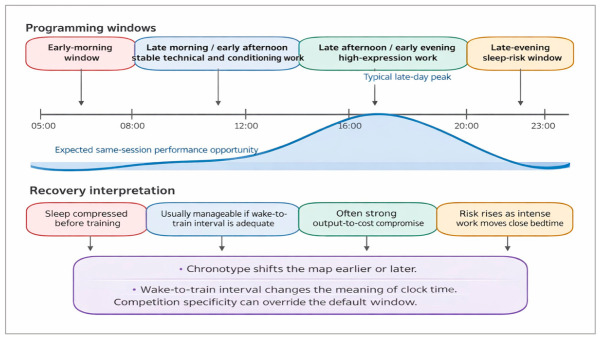
Conceptual chrono-aware 24 h map for session placement. Performance opportunity usually rises from late morning to early evening, whereas biological cost increases when training compresses sleep before an early start or extends too close to bedtime. Chronotype, wake-to-training interval, and competition demands shift the practical position of each window [6,8,9,11,12,13,14,37,38,39,40,41,46,47,48,75,84,85,88].

Importantly, training at competition clock time after travel is not always the first priority. In some cases the athlete first needs circadian stabilization and sleep protection before high-quality work is reintroduced. The biological logic is identical to the performance-to-cost framework developed in this review: a session that looks specific on the calendar may still be mistimed if it occurs at the wrong internal phase or after acute sleep debt [45,46,47,48]. This trade-off across the 24 h cycle is illustrated in Figure 2.

## 7. Redox-Inflammatory and Endocrine Responses

Training adaptation requires stress signaling, not an absence of stress. What matters is the amplitude, timing, tissue specificity, and recovery context of those signals. Circadian biology is relevant because redox state, immune trafficking, hormonal milieu, and mitochondrial behavior all vary across the day, meaning the same external load can arrive on a different molecular background [19,20,61,93]. However, the mechanistic resolution of this section is not uniform across evidence domains: the strongest athlete-relevant evidence remains human and performance-oriented, whereas many redox-inflammatory and clock-pathway insights still derive from preclinical models or clinical populations.

### 7.1. Molecular Clockwork, Energetic Sensing, and Mitochondrial Readiness

At the mechanistic level, BMAL1:CLOCK interacts with SIRT1, AMPK, PGC-1alpha, NAD+ availability, mitochondrial biogenesis, substrate oxidation, and transcriptional flexibility [19,20,61,65,68,94]. This supports a plausible phase-dependent signaling hierarchy, but the translational weight is uneven: pathway-level evidence is strong in experimental models, while athlete-specific molecular confirmation remains sparse. Therefore, the molecular section should be read as a mechanistic explanation for why timing may matter, not as proof that one clock hour is universally superior for athletes.

Human evidence is strongest where it shows rhythmic or clock-driven programs in skeletal muscle, altered metabolic signatures under circadian misalignment, and training-related modification of clock gene expression in metabolically altered cohorts [26,54,97,100,101,102,103,104]. These findings justify studying exercise timing in athletic tissue and field contexts, but they do not yet define a direct molecular prescription for sport. Preclinical BMAL1/CLOCK and timed-exercise models are retained here only to identify candidate mechanisms, including mitochondrial readiness, substrate selection, and repair-versus-stress signaling [62,63,64,65,66,67,68,69,70,71,72,106,107,108,109].

### 7.2. Redox-Inflammatory Timing and Recovery Cost

Redox signaling is likewise not time-neutral. Exercise-generated reactive oxygen species can serve as eustress signals that stimulate adaptation, but the downstream consequence of a given redox impulse likely depends on baseline antioxidant capacity, mitochondrial state, and clock-regulated defenses such as NRF2-linked programs [68,93,110]. This is relevant because circadian disruption and muscle clock dysfunction are associated with impaired oxidative defense, altered myokine release, fibrosis, and reduced tissue resilience [66,93].

Direct human evidence for redox-inflammatory timing in sport remains limited. The most defensible interpretation is that time of day may change the recovery cost of a given load through baseline antioxidant capacity, mitochondrial state, sleep proximity, endocrine context, and immune rhythm, but the size and direction of this effect in trained athletes are still uncertain. Human eccentric exercise and metabolomic/proteomic studies should therefore be prioritized, whereas mouse transcriptomic studies are best framed as hypotheses about repair-oriented versus stress-associated signaling that require athlete verification [26,54,72,110].

### 7.3. Endocrine Context, Sleep Window, and Sampling Design

Endocrine context adds another layer. Morning exercise is superimposed on high cortisol and a distinct autonomic background, whereas late-day exercise occurs in a different balance of sympathetic tone, temperature, and feeding history [2,5,61]. These conditions can modify insulin sensitivity, substrate partitioning, and perceived strain without implying that one clock time is inherently anabolic or catabolic in all settings [61,65,71].

Because endocrine and adaptive responses are conditioned by substrate availability, selected nutrition and supplement strategies cannot be separated completely from training time. In practice, the acute response to resistance-oriented or hypertrophy-oriented work is likely shaped by both clock phase and the composition/timing of peri-exercise nutrition, especially when energy availability is constrained or when protein distribution across the day is poorly aligned with the training stimulus [55,83,118].

A final methodological point is critical: molecular sampling must respect time. Immediate post-exercise samples capture signaling initiation; delayed samples capture downstream inflammatory, mitochondrial, endocrine, and epigenetic remodeling. Because several biomarkers themselves oscillate across the day, chrono-exercise studies need repeated post-exercise sampling, fixed wake times, controlled meals/light/caffeine, and next-day functional outcomes if they want to interpret biology rather than merely describe a snapshot [27,54,72,93,105,110,119,120,121]. This is especially important when investigators want to distinguish a beneficial adaptive signal from a high-cost state characterized by sleep loss, metabolic misalignment, or residual overload.

### 7.4. Sex-Specific Context and Female-Athlete Considerations

Chrono-exercise evidence remains male-dominated, which limits external validity for female athletes. Available data suggest that morning–evening differences in neuromuscular function may not be identical across sexes, while broader circadian literature shows sex-related differences in sleep timing, waking cognition, thermoregulation, and endocrine signaling [111,112,113,114,115].

Interpretation therefore requires contextual reporting rather than generic extrapolation. Menstrual-cycle phase, hormonal contraceptive use, symptom burden, sleep quality, and thermoregulatory strain can each alter readiness or recovery and may amplify or mask apparent timing effects [58,115,116,117]. When these variables are omitted, it becomes difficult to determine whether the observed difference is primarily circadian, endocrine-contextual, or both.

For practice, the safest conclusion is individualization. Coaches should avoid assuming that evidence derived mostly from male cohorts transfers unchanged to female athletes, particularly when interpreting fatigue, mood, thermoregulation, and sleep responses to early or late sessions.

## 8. From Chronobiology to Periodization: A Practical Sport Science Framework

The simplest applied synthesis is that session timing should be selected according to the objective of the session, the chronobiological profile of the athlete, and the recovery demands of the surrounding microcycle. A useful coaching heuristic is to seek the best output-to-cost ratio for the planned stimulus rather than the highest same-session output at any price. In this review, that logic is formalized as the Performance–Biological Cost (PBC) model: a systems-level framework in which training time functions as a dynamic control variable within the broader adaptation-recovery architecture. This framing is conceptually aligned with recent systems views of load oscillation, energy status, and recovery cost across the overload–recovery continuum [119,122,123,124].

For practical application, the Performance–Biological Cost (PBC) framework can be approximated as: PBC ≈ Performance Output/Biological Cost, where performance output reflects external work and task quality, while biological cost integrates internal load, autonomic disturbance, sleep disruption, and residual molecular stress.

When a session is designed to maximize speed, power, or high-quality repeated efforts, late afternoon or early evening is often an attractive default because it aligns with the time window in which neuromuscular performance is frequently highest [6,8,9]. However, if the sport competes regularly in the morning, then at least part of the preparation should deliberately expose athletes to morning high-quality work, otherwise the program may optimize for the wrong competitive reality [3,10].

Sessions with a potentially large autonomic or sleep cost should be placed with particular care. A high-intensity session completed too close to bedtime may offer good immediate output yet compromise nocturnal recovery, whereas a very early session may save the evening but compress sleep beforehand. Coaches should therefore judge session timing across the whole 24 h cycle, not only the training block itself [11,12].

For applied programming, the following ranges can be used as starting anchors and then individualized: low- to moderate-intensity morning work may be placed 30–90 min after waking if sleep is preserved; maximal-strength, sprint, or HIIT sessions in the morning should preferably occur after about 90–180 min of wakefulness with deliberate warm-up and fueling; vigorous evening sessions should generally finish at least about 2–3 h before intended sleep when recovery is the priority; and after major travel or late-night competition, the first 24–48 h should prioritize sleep stabilization, light exposure, and low-cost re-entrainment before demanding high-quality work. These ranges are deliberately conservative because the goal is not to maximize output once, but to preserve the output-to-cost ratio across the microcycle.

Table 5 translates the review into a session-objective matrix. The purpose is not to rigidly assign one perfect hour, but to make explicit when late-day quality is desirable, when competition-specific morning exposure should dominate, and when biological cost should overrule acute output.

To facilitate practical application of the PBC framework across different athlete profiles and scheduling contexts, a decision-oriented synthesis is presented in Table 6.

In practice, a circadian-aware periodization model can be summarized in six operational principles:

- Place high-expression speed-power or demanding small-sided game sessions at a time of day that supports quality, commonly late afternoon or early evening when schedules permit.
- Use regular, purposeful morning exposures when competition, travel, or environmental realities require morning performance rather than assuming that a better afternoon response will transfer automatically.
- Avoid placing very intense or high-volume sessions unnecessarily close to bedtime; when sleep protection is a priority, use a conservative finish-to-sleep buffer of about 2–3 h for HIIT, repeated-sprint, or heavy resistance work [11].
- Protect morning sessions with sufficient sleep opportunity, early nutritional planning, light exposure where appropriate, and a deliberate warm-up; for maximal morning sessions, a 90–180 min wake-to-training interval is a useful starting anchor when logistics permit.
- Interpret timing decisions through a combined monitoring lens that includes external output, internal load, subjective recovery, HRV trends, sleep metrics, mood, soreness, and next-day skill or performance quality [13,22].
- Where possible, individualize the wake-to-training interval, session-to-sleep interval, caffeine timing, light exposure, hydration, and thermal preparation rather than relying only on the labels morning and evening.

These principles are deliberately modest. They do not require expensive molecular assays to become useful. Simple sport-science monitoring—training performance, session RPE, mood, sleep quantity, and next-day readiness—can already reveal whether a specific timing strategy is working for a given athlete or squad. Where morning quality work or aggressive recovery are priorities, timing decisions should also be coordinated with peri-exercise energy support rather than treated independently from the nutrition plan [82]. The molecular language of redox balance, inflammation, mitochondrial remodeling, and autonomic recovery strengthens the conceptual model, but the daily-applied test remains practical: did the chosen timing improve training quality without imposing an unsustainable cost?

**Figure 3:**
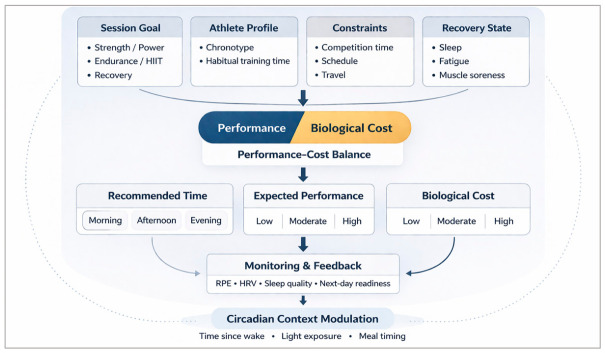
Applied decision model for training-time selection based on the Performance–Biological Cost (PBC) framework. Circadian-informed decision framework integrating session objectives, athlete profile, contextual constraints, and recovery state to optimize training time based on the balance between performance and biological cost, with continuous feedback from monitoring variables (e.g., RPE, HRV, sleep, next-day readiness) [3,6,8,9,10,11,12,13,14,22,25,42,75,88,89,90,91,92,119,122,123,124].

The conceptual principles outlined above can be operationalized into a structured decision-making framework that integrates performance objectives, athlete characteristics, contextual constraints, and recovery dynamics. In this approach, training time is treated as a dynamic control variable rather than a fixed scheduling choice, optimized through the balance between expected performance and associated biological cost. This logic is formalized in the applied PBC-based decision model presented in Figure 3.

Figure 3 conceptualizes training-time placement as an iterative control process rather than a static scheduling decision. By explicitly linking performance output to its associated biological cost, the framework allows practitioners to evaluate not only when performance is maximized, but also when it is most sustainable within the broader recovery architecture. In this sense, training time becomes a tunable variable within periodization, continuously adjusted based on monitored responses rather than predetermined by clock time alone.

In applied settings, PBC can be operationalized using combined monitoring variables such as external load metrics, session RPE, heart rate variability, sleep indices, and next-day readiness.

### Testable Predictions Emerging from the Framework

Based on the integrative performance-to-biological-cost framework proposed in this review, several testable predictions can be formulated to guide future experimental and applied research:

**P1.** Output–cost dissociation across the day—For equivalent external load, late-afternoon or early-evening high-intensity exercise is expected to produce higher performance output but also a greater acute biological cost when scheduled too close to the sleep window, detectable via lower same-night HRV, higher nocturnal heart rate, longer sleep latency, or larger next-morning inflammatory/redox perturbation [11,35,36,37,38,39,110].

**P2.** Chronotype and wake-to-training interaction—The magnitude and direction of phase-dependent effects on performance and internal load will be strongly modulated by chronotype and wake-to-training interval, such that early sessions performed within the first few hours after waking in evening-type individuals will disproportionately increase internal load relative to performance output, sleepiness, and self-reported readiness [42,75,84,85,87].

**P3.** Time-specific adaptation and entrainment—Repeated training at a fixed time of day will induce time-specific improvements in performance and associated molecular signaling, reflected in trained-time improvements, clock gene remodeling, and phase-sensitive substrate or mitochondrial signatures, without necessarily conferring a universal performance advantage across all time points [26,54,97,100,108,109,125].

These predictions provide a basis for future studies integrating performance, recovery, and molecular outcomes, particularly in ecologically valid athletic settings characterized by high training loads and constrained recovery opportunities. A concise applied implementation sequence is summarized in Box 1.

**Box** **1** **Applying the Performance–Biological Cost (PBC) Framework.**

 **Step 1: Define the objective.** Performance expression, conditioning, technical-tactical quality, or recovery?

 **Step 2: Estimate expected output.** Consider circadian phase, chronotype, habitual training time, and wake-to-training interval.

 **Step 3: Estimate biological cost.** Consider session RPE, HRV suppression, sleep interaction, residual fatigue, and next-day readiness.

 **Step 4: Optimize the ratio.** Choose the training time that maximizes output relative to cost.

 **Step 5: Update with monitoring.** Use performance trends, recovery, mood, sleep, and next-day readiness.

 **Key principle.** Judge timing by the best balance between output and sustainable biological cost across the microcycle.

## 9. Methodological Limitations and Future Directions

The chrono-exercise literature is informative but still limited by recurring methodological weaknesses. Many studies compare only two clock times without reporting chronotype, habitual training time, preceding sleep, meal timing, caffeine intake, light exposure, hydration, environmental temperature, wake-to-training interval, or session-to-sleep interval. These omissions are not trivial because each can shape both performance and the biological meaning of a session. The present review also remains limited by its narrative design: no preregistered protocol, PRISMA flow diagram, formal risk-of-bias scoring, or new meta-analysis was performed, so the stability of pooled effects must be inferred from existing meta-analyses rather than recalculated here.

A second limitation is heterogeneity of the evidence base. The review integrates elite athletes, team sport players, healthy active adults, clinical or metabolically altered cohorts, and animal or cellular models. This broad scope is useful for building the PBC framework, but it mixes populations with different training status, sleep routines, metabolic phenotypes, sex-specific contexts, and recovery demands. Consequently, clinical human and preclinical findings were used to explain plausible mechanisms, whereas evidence from human athletes was weighted most heavily for prescription.

A third limitation is the frequent overemphasis on acute output without adequate recovery follow-up. A study that reports higher evening power but no information on night-time sleep, nocturnal heart rate, HRV trend, next-morning readiness, or subsequent training quality provides only part of the answer. Equally, single-point biomarker sampling may miss delayed inflammatory, mitochondrial, endocrine, or epigenetic responses. The field therefore needs designs that connect the session to the next day and to the surrounding microcycle, not only to the minute after exercise.

Female athletes remain underrepresented, and menstrual-cycle phase, hormonal contraceptive use, symptom burden, thermoregulation, and sleep vulnerability are still inconsistently addressed. Team sport ecology is also insufficiently captured when laboratory tests are translated too quickly into practical advice. Real calendars include technical, tactical, academic, occupational, travel, media, and competition-density constraints that can alter the optimal placement of sessions and can confound apparent timing effects.

Future work should move beyond simplistic clock time comparisons. What matters in practice may be the interval between wake time and training, the interval between training and sleep, the interaction with chronotype, objective circadian phase, muscle clock phase, competition schedule, and the cumulative architecture of two-a-day or congested schedules. Studies that combine wearable-derived sleep and HRV data with field performance, repeated biomarker sampling, and selected molecular markers would be especially valuable because they would connect mechanism to real program design.

An immediate opportunity is to connect field wearables with repeated biological sampling. Actigraphy, nocturnal HRV, body temperature, ecological momentary ratings, continuous glucose monitoring, and selected blood or muscle markers could be integrated to determine whether a timing strategy truly improves the output-to-cost ratio [41,42,83,119]. This approach would help move the field beyond simple clock-time comparisons toward phase-aware models that incorporate wake timing, light history, meal timing, travel, sex-specific context, and interpretable decision support.

Rather than prescribing a formal reporting standard, the following checklist is intended as a pragmatic guide to improve interpretability, comparability, and ecological validity in future chrono-exercise research.

A critical next step for the field is to move from descriptive time-of-day comparisons toward integrative models that quantify the interaction between performance, recovery, and biological cost across the 24 h cycle.

Table 7 translates these recurring design issues into a practical checklist for future studies.

## 10. Conclusions

Time of day should no longer be treated as a peripheral scheduling detail in sport science, but as a meaningful programming variable that modulates both performance expression and the biological cost of training. The circadian-metabolic state in which exercise is performed shapes not only acute output, but also how that load is internally processed, recovered from, and translated into adaptation.

Current evidence indicates that while late-day training often favors neuromuscular performance, there is no universally optimal training time for long-term adaptation. Instead, identical external loads may carry different physiological and molecular costs depending on temporal placement, chronotype, wake-to-training interval, session-to-sleep interval, competition calendar density, travel, and the degree of training–testing congruency.

From a practical standpoint, training time should be recognized as a hidden load variable within periodization. A session performed at an unfavorable circadian phase may achieve the desired external output, but at a disproportionately high biological cost, contributing to accumulated fatigue, impaired recovery, and reduced adaptation efficiency.

Accordingly, the key question is not when performance is maximized, but when performance can be achieved at the lowest sustainable cost. Within this framework, exercise timing becomes a dynamic control variable, shaping not only how athletes perform, but how effectively they adapt.

## Figures and Tables

**Table 1:** Evidence architecture and representative characteristics of the literature used in the structured narrative synthesis.

Evidence Tier	Typical Designs and Populations	Timing/Context Variables Extracted	Function in the Synthesis
Meta-analyses and systematic reviews	Human syntheses on endurance, strength, short-duration maximal exercise, repeated sprinting, team-sport performance, chronotype, sleep, HRV, and travel [4,6,7,8,9,22,23,24,25].	Clock-time comparison, outcome domain, effect direction or magnitude, heterogeneity, chronotype or habitual-timing modifiers when reported.	Anchor the direction and approximate magnitude of acute performance, sleep, recovery, and chronotype-related conclusions.
Randomized or crossover human trials	Controlled acute or short-term studies in healthy, trained, or metabolically altered adults, including resistance, endurance, HIIT, and evening-exercise designs [11,26,27,28,29,30,31,32,33,34,35,36,37,38,39,40,41].	Specific clock times, feeding status, exercise mode, sleep window, internal load, HRV, metabolomic/proteomic or clock gene readouts.	Provide mechanistic or causal human support, but with caution when populations are not athletes or protocols are laboratory constrained.
Athlete field and competition studies	Elite or competitive sport datasets, including soccer training, Olympic-level outcomes, professional travel/circadian-misalignment studies, and athlete sleep cohorts [13,42,43,44,45,46,47,48,49,50].	Competition time, training time, travel history, days since arrival, match density, mood, well-being, technical quality, sleep, and readiness.	Supply ecological validity and identify calendar, travel, and competition-specific confounders that laboratory studies may miss.
Time-specific training interventions	Morning-versus-evening or trained-time studies evaluating chronic adaptation, detraining, or testing congruency [3,10,32,51,52,53,54].	Training hour, testing hour, habitual training time, duration of intervention, performance transfer across time points.	Inform the distinction between acute peak expression and long-term adaptation at the trained or target competition time.
Human mechanistic and tissue-level studies	Human skeletal muscle clock, transcriptomic, metabolomic, insulin-sensitivity, and circadian misalignment studies [9,10,11,12,13,14,15,16,17,18,19,20,21,22,23,24,25,26,27,28,29,30,31,32,33,34,35,36,37,38,39,40,41,42,43,44,45,46,47,48,49,50,51,52,53,54,55,56,57,58,59,60,61,62,63,64,65,66,67,68,69,70,71,72,73,74,75,76,77,78,79,80,81,82,83,84,85,86,87,88,89,90,91,92,93,94,95,96,97,98,99,100,101,102,103,104,105].	Sampling time, phase markers, metabolic phenotype, clock gene expression, mitochondrial or substrate-use signatures.	Support biological plausibility while requiring careful translation before athlete prescription.
Preclinical and cellular models	Mouse or cell-based clock-disruption, timed-exercise, redox, mitochondrial, and BMAL1/CLOCK studies [62,63,64,65,66,67,68,69,70,71,72,93,106,107,108,109].	Zeitgeber time, genetic model, tissue specificity, transcriptomic/proteomic response, repair or stress pathways.	Used only as mechanistic scaffolding and to generate testable athlete hypotheses, not as direct evidence for an optimal training hour.

Notes: HIIT, high-intensity interval training; HRV, heart rate variability. The table is intended to make the narrative selection logic auditable without converting the article into a formal systematic review.

**Table 2:** Evidenceonfidence map for the main chrono-exercise claims used to build the Performance–Biological Cost (PBC) framework.

Domain or Claim	Direction and Approximate Magnitude	Evidence Confidence	Main Modifiers and PBC Interpretation
Acute neuromuscular output	Late-afternoon/evening advantages are recurrent; effects are often small-to-moderate and larger for Wingate power and jump height than for some endurance outcomes [6,8,9,24].	High to moderate	Chronotype, habitual training time, warm-up, temperature, and testing congruency determine whether higher output is achieved at acceptable biological cost.
Endurance performance	Late-day advantages are common but heterogeneous; effects depend on test duration, pacing, feeding, thermal load, and prior sleep [6,7].	Moderate	Morning underperformance should not be interpreted as purely circadian until sleep, fueling, caffeine, and warm-up have been controlled.
Chronic adaptation	No universal optimal hour is supported; trained-time improvements and training–testing congruency are more consistent than global morning/evening superiority [3,10,27,32,51,52,53].	Moderate	The relevant target is adaptation at the competition-relevant phase, not only peak expression during a laboratory test.
Internal load, mood, and skill quality	Evidence is thinner than for output, but field studies indicate that timing can influence well-being and high-intensity or technical responses [43].	Low to moderate	A time slot may be favorable if it preserves mood, engagement, and next-session quality even when acute output differences are modest.
Sleep and autonomic recovery	Evening exercise is not uniformly harmful, but vigorous exercise close to bedtime and very early training after curtailed sleep can raise biological cost [11,12,13,14,25,35,36,37,38,39,40,41,88,89,90,91,92].	Moderate for sleep; low to moderate for HRV	Session-to-sleep and wake-to-training intervals should be treated as programming variables rather than ignored confounders.
Molecular timing and redox-inflammatory pathways	Human evidence suggests time-sensitive metabolomic, proteomic, and clock gene responses; preclinical studies provide stronger causal but indirect support [26,54,70,71,72,73,93,97,100,108,109,110].	Low to moderate for athletes	Molecular claims should be framed as plausible mechanisms and testable predictions, not as direct athlete prescriptions.
Competition scheduling and travel	Competition outcomes and professional sport data indicate meaningful circadian and travel-related effects, but calendar density can confound interpretation [44,45,46,47,48,78,79,80].	Moderate	The same clock time may carry different costs depending on fixture density, travel direction, days since arrival, and next competition.
Sex-specific context	Evidence remains male dominated, with insufficient control of menstrual-cycle phase, hormonal contraception, thermoregulation, and sleep vulnerability [111,112,113,114,115,116,117].	Low	Individual monitoring is preferable to direct transfer of male-derived timing rules to all female athletes.

Notes: Confidence categories are pragmatic and narrative, not a formal GRADE rating. They summarize directness, consistency, population relevance, and ecological validity across the cited literature.

**Table 3:** Athlete- and context-level modifiers of the apparent morning-evening effect.

Athlete or Context	Likely Source of Altered Response	Programming Implication	Monitoring Priority
Morning chronotype or habitual early trainer	Smaller subjective and performance penalty in early sessions; faster alignment to morning competition.	Morning quality work is more tolerable, but still protect sleep and warm-up.	Sleep duration, wake-to-train interval, session RPE
Evening chronotype or habitual late trainer	Greater early-day decrement in alertness, mood, and output; larger mismatch when forced to train early.	Retain some morning exposure for specificity, but do not overload the athlete with repeated high-cost AM quality sessions.	Mood, perceived readiness, next-day fatigue
Morning-competition athlete	The target event occurs when temperature, arousal, and wake history may not yet favor peak expression.	Use deliberate morning rehearsal blocks and protect the pre-competition sleep period.	Competition-specific output, sleep opportunity
School or work-constrained team-sport athlete	External schedule may dominate over biological preference, inflating internal load or reducing technical quality.	Match the most cognitively and neuromuscularly demanding content to the best feasible window, not simply the only available slot.	Skill quality, mood, attendance, soreness
Double session or congested microcycle	A session that improves same-day output may still increase biological cost before the next key practice.	Judge timing across the whole 24 h cycle and preserve the highest-value session of the day.	HRV, sleep, morning readiness
Long-haul travel/ jet lag	Local clock time is decoupled from internal phase, sleep, and feeding rhythms.	Prioritize re-entrainment and sleep stabilization before demanding high-quality work.	Sleep timing, daytime sleepiness, perceived freshness
Female-athlete or sex-specific endocrine context	Timing responses may be shaped by underreported endocrine, thermoregulatory, and sleep-related factors.	Avoid one-size-fits-all conclusions; interpret timing together with hormonal and symptom context when relevant.	Sleep, symptoms, performance trend, perceived exertion
Congested competition calendar or tournament block	Fixture density, repeated travel, residual muscle damage, emotional arousal, altered tapering, and reduced sleep opportunity can mask or amplify apparent time-of-day effects.	Interpret morning/evening responses relative to days since last competition, next-start time, travel direction, and accumulated match or race load rather than clock time alone.	Match minutes or race load, days since competition, soreness, sleep debt, HRV/readiness, and next-start timing

Notes: AM, morning; RPE, rating of perceived exertion. The table synthesizes evidence on chronotype and time-specific training [4,10,51,52,53,75,84,85,86,87], sleep/recovery constraints [11,12,13,14,22], athlete field context [9,13,42,43,44], and travel-related circadian misalignment [45,46,47,48,78,79,80]; entries are intended as decision aids rather than rigid rules.

**Table 4:** Evidence domains, candidate pathways, and applied implications for training-time decisions in sport.

Evidence Domain	Typical Finding	Key Modifiers, Mediators, and Pathways	Practical Implication
Neuromuscular output	Late-afternoon/evening advantages are frequent for maximal strength, jump, sprint, and repeated-sprint tasks.	Body temperature, neural readiness, chronotype, warm-up quality, habitual training time, and muscle-clock phase.	Use late afternoon/evening for high-expression speed-power work when schedules allow, but retain morning exposure when competition requires it.
Endurance tasks	Afternoon/evening advantages are common but smaller and less uniform than for explosive tasks.	Feeding status, prior sleep, pacing, thermal load, glycogen availability, and phase-sensitive metabolic signaling.	Do not treat morning performance as fixed; protect sleep, fueling, and warm-up before concluding inferiority.
Team-sport training quality	Field data suggest session timing can influence mood, technical quality, and high-intensity actions.	Training content, athlete buy-in, decision load, chronotype mismatch, and recovery context.	Match demanding tactical or speed-based work to the window that best supports quality and engagement.
Autonomic recovery	Evidence is limited, but post-exercise autonomic disturbance and parasympathetic reactivation may vary with timing and chronotype.	Exercise intensity, chronotype mismatch, sleep proximity, prior fatigue, and circadian-autonomic coupling.	Interpret output together with HRV, perceived recovery, and next-day readiness.
Sleep interaction	Early sessions may reduce sleep opportunity, whereas late vigorous sessions can impair nocturnal recovery when too close to bedtime.	Wake time, travel, session-to-sleep interval, evening exercise dose, and thermoregulatory/endocrine context.	Theoretical performance gains should not repeatedly come at the cost of compromised sleep.
Chronic adaptation and molecular timing	Time-specific training improves performance at the trained hour, but universal superiority of one clock time is not supported.	Training–testing congruency, muscle-clock entrainment, clock gene remodeling, and AMPK-SIRT1-PGC-1alpha signaling.	Program timing according to season goals, competition schedule, and desired output-to-cost ratio.
Chronotype and wake-to-train interval	The same wall-clock hour can be highly favorable in one athlete and strongly constraining in another, especially soon after waking.	Chronotype, sleep inertia, habitual wake time, prior light exposure, and time since waking.	Individualize morning exposure instead of assuming that all athletes experience the same morning penalty.
Travel and circadian misalignment	Performance and recovery can deteriorate when local time, internal phase, and competition time are misaligned.	Direction of travel, light management, sleep debt, meal timing, and re-entrainment strategy.	After travel, prioritize circadian stabilization and sleep protection before demanding high-quality work.
Sex-specific context	Evidence remains male dominated, and female-athlete responses may be shaped by endocrine and sleep-related context that is often underreported.	Menstrual-cycle phase, hormonal contraceptive use, sleep, thermoregulation, and sex differences in circadian organization.	Avoid assuming direct transfer of male-derived timing data to all female athletes; monitor individually.
Co-zeitgebers: light, meals, and caffeine	Part of an apparent time-of-day effect may reflect pre-session light history, fueling status, caffeine timing, or late-night circadian disruption rather than clock time alone.	Bright light, melatonin suppression, breakfast timing, carbohydrate availability, and caffeine strategy.	Treat light and nutrition as controllable levers that can reduce unnecessary biological cost.
Competition calendar ecology	Intensive competition schedules can obscure the true timing effect by changing fatigue state, sleep opportunity, travel alignment, and tactical or emotional load.	Fixture density, kickoff/event time, days since last competition, days to next competition, taper status, and time-zone history.	Report calendar density and treat it as a confounder or modifier when interpreting time-of-day effects in athletes.

Notes: HRV, heart rate variability; AMPK, AMP-activated protein kinase; SIRT1, sirtuin 1; PGC-1alpha, peroxisome proliferator-activated receptor gamma coactivator 1alpha. Practical implications reflect synthesis of evidence on acute performance [6,7,8,9,24], chronotype and timing congruency [10,22,23,44,51,52,53,75,84,85,86,87], molecular timing mechanisms [17,19,20,26,54,62,63,64,65,66,67,68,69,70,71,72,73], sleep/autonomic recovery [11,12,13,14,22,23], and circadian modifiers such as light, meals, and travel [42,45,46,47,48,55,78,79,80,81,82,83]; they indicate the default choice most often supported by the surrounding evidence, not a universal prescription.

**Table 5:** Session-objective matrix for chrono-aware session placement across the microcycle.

Session Objective	Usually Favorable Window	Reasons to Override the Default	Protective Measures and Key Readouts
Max strength, power, sprint, repeated efforts	Late afternoon to early evening is often favorable for peak expression.	Morning competition, travel, or a need to rehearse early performance may outweigh raw output.	Warm-up quality; sleep protection; if performed in the morning, preferably allow about 90–180 min after waking plus breakfast/caffeine strategy before maximal expression; output plus session RPE and next-day readiness.
Aerobic base, tempo, moderate conditioning	Stable daytime is often a practical compromise between readiness and schedule.	Environmental heat, fuel availability, or double-session structure may justify earlier or later placement.	Fueling, hydration, thermal stress, and the effect on the following key session.
Threshold or HIIT with high recovery cost	Place where output is acceptable without compressing sleep or compromising the next training day.	Very late completion near bedtime or very early starts after curtailed sleep should be avoided when possible.	Nocturnal sleep, morning HRV or perceived freshness, residual soreness; for vigorous HIIT, aim to finish at least about 2–3 h before attempted sleep when possible.
Small-sided games or demanding tactical work	Use the window that best supports combined neuromuscular freshness and cognitive engagement.	Facility logistics, academic constraints, and match-specific rehearsal may modify the choice.	Technical quality, decision speed, mood, and willingness to sustain intensity.
Competition-specific morning rehearsal	Strategic rather than constant use; rehearse the target competitive reality.	Not every quality session should be forced into the morning if the biological cost becomes excessive.	Wake-to-train interval, breakfast timing, warm-up, and day-level fatigue; rehearse the target start time with protected sleep and a consistent pre-event routine.
Recovery, re-entrainment, or post-travel day	Default to the least disruptive slot that restores rhythm and sleep opportunity.	After major travel or late arrival, circadian stabilization may matter more than sport-specific clock time.	Sleep timing, daytime sleepiness, appetite rhythm, low-intensity tolerance.

Notes: HIIT, high-intensity interval training; HRV, heart rate variability; RPE, rating of perceived exertion. The table synthesizes evidence on diurnal performance and time-specific training adaptation [3,6,8,9,10,24,51,52,53], sleep-autonomic recovery constraints [11,12,13,14,22,25,35,36,37,38,39,40,41,88,89,90,91,92], team-sport session quality [43], travel-related circadian misalignment [42,46,47,48,78,79,80], and systems views of training cost and recovery [119,122,123,124]. “Usually favorable window” denotes the option most often supported by the current evidence when no overriding competition or recovery constraint is present.

**Table 6:** Decision matrix for session timing according to chronotype, competition demands, and recovery constraints.

Context	Morning Placement	Afternoon/ Evening Placement	PBC Decision Rule
Morning chronotype + morning competition	Use regular high-quality rehearsal at the target start time. For maximal sessions, protect sleep and aim for about 90–180 min wake-to-training when feasible.	Retain some late-day quality work for overload, but do not rely on it alone for competition specificity.	Prioritize congruency with the competitive phase while monitoring whether morning quality carries excessive next-day cost.
Evening chronotype + morning competition	Introduce morning exposure progressively. Begin with technical/low-cost work, then add key sessions once sleep, breakfast, light, caffeine, and warm-up routines are stable.	Use late-day sessions for peak output when the calendar allows, but avoid overprotecting the athlete from the required morning state.	Use the lowest morning dose that creates specificity without chronic sleep compression or inflated session RPE.
Morning chronotype + late-day competition	Morning work is useful for base, recovery, or secondary sessions, especially if it protects evening freshness.	Place decisive speed-power, tactical, or competition-specific work late day when sleep is not compromised.	Exploit chronotype tolerance for morning support work while preserving the competition-relevant window for expression.
Evening chronotype + late-day competition	Avoid unnecessary early high-cost work unless logistics require it; use morning for recovery, mobility, or low-intensity conditioning.	Late-day quality is often biologically and psychologically favorable, but vigorous work should finish about 2–3 h before sleep when recovery is critical.	This is often the best output window, but the session-to-sleep interval remains the limiting cost variable.
Congested fixtures or two-a-day microcycle	Use morning only if it does not reduce sleep below the athlete’s normal requirement; favor low-cost technical or aerobic content after late competition.	Late-day output may be higher, but it can interfere with sleep or the next key session if placed too late.	Choose the slot that protects the highest-value session of the next 24–48 h, not the slot that maximizes today’s isolated output.
Travel, jet lag, or late arrival	Early local-time training may be useful only after sleep and light strategy are stabilized; otherwise use low-intensity re-entrainment.	Afternoon sessions can help adaptation, but avoid mistimed hard work when the athlete is still internally misaligned.	Treat internal circadian phase, days since arrival, and sleep debt as stronger constraints than local wall-clock time.

Notes: The ranges in this table are pragmatic anchors derived from sleep, chronotype, time-specific training, and athlete travel literature; they should be individualized by monitoring sleep, RPE, HRV trends, mood, and next-day task quality.

**Table 7:** Methodological considerations for chrono-exercise research in athletes and physically active adults.

Variable	Rationale	Recommended Practice
Chronotype	The same clock hour is not biologically identical across athletes.	Report chronotype questionnaire data, but where feasible add objective phase markers such as DLMO, actigraphy-derived sleep timing, or continuous temperature rhythm; explore whether responses differ between morning- and evening-oriented participants.
Habitual training time	Repeated exposure can attenuate diurnal performance differences and alter local clock gene expression.	Report the usual training schedule and the degree of mismatch between habitual and experimental timing.
Wake-to-training interval	Clock time alone is incomplete; readiness also depends on time since waking and residual sleep inertia.	Report wake time and the interval between waking and session start.
Sleep opportunity and session-to-sleep interval	Early sessions may reduce sleep, whereas late sessions may impair the pre-sleep window.	Report bedtime, wake time, sleep duration, and the interval between session end and attempted sleep.
Meals, hydration, and caffeine	Morning deficits may partly reflect incomplete fueling, hydration status, or stimulant use rather than circadian biology alone.	Standardize pre-session meals, hydration, and caffeine timing across conditions.
Warm-up design	A stronger warm-up can reduce part of the morning disadvantage.	Describe and standardize warm-up duration, intensity, and thermal content.
Sex and hormonal context	Hormonal status may influence both performance and recovery, yet female athletes remain underrepresented.	Report sex clearly and, where relevant, describe menstrual-cycle or hormonal-contraceptive considerations.
Internal plus external load and next-day recovery	Output alone does not describe the cost of the session.	Pair performance with RPE, heart rate, mood, soreness, HRV or sleep metrics, and next-day readiness where feasible.
Biomarker and omics sampling window	Immediate and delayed responses may tell different stories, especially after damaging exercise; light exposure should also be standardized when possible.	Use repeated post-exercise sampling for molecular outcomes and report light exposure or other zeitgebers when relevant.
Light exposure and other zeitgebers	Light history, screen exposure, and meal timing can shift circadian phase or modify acute readiness independent of exercise itself.	Report pre-session light exposure and any deliberate light, melatonin, or meal-timing intervention.
Travel, time zone, and local clock alignment	A session performed at local clock time may occur at a very different internal phase after travel.	Report recent travel, time-zone change, arrival time, and days since arrival.
Environmental temperature and thermal preparation	Some morning–evening differences are partly mediated by temperature and can be masked or exaggerated by heat or passive warming.	Report ambient conditions and any passive heating, cooling, or thermal-maintenance strategy.
Competition specificity and testing congruency	The trained hour and the tested hour should not be treated as interchangeable, especially in applied sport.	State explicitly whether the design evaluates acute peak output, competition-specific rehearsal, or chronic adaptation at a target clock time.
Objective circadian phase markers	Questionnaires such as morningness–eveningness instruments capture preference, not necessarily biological phase; athlete schedules can distort questionnaire-phase agreement.	When feasible, include DLMO, core or distal temperature rhythm, actigraphy, or validated sleep-timing markers, especially in elite, traveled, or irregularly scheduled cohorts.
Competition calendar density	Recent competition, congested fixtures, early heats, late finals, and broadcast-driven start times can alter sleep, residual fatigue, and readiness independently of circadian phase.	Report days since last competition, days to next competition, event start time, match minutes or race load, taper status, and travel history over at least the preceding 7–14 days.
HRV interpretation	HRV is sensitive to hydration, heat, illness, emotional stress, menstrual symptoms, sleep loss, and residual sympathetic activation; isolated values can be misleading.	Interpret HRV as a trend alongside sleep, perceived recovery, soreness, session RPE, and performance rather than as a stand-alone biological-cost marker.
Evidence-level transparency	Narrative reviews risk selective emphasis when study inclusion and evidence hierarchy are not made explicit.	State the inferential role of each evidence tier and distinguish athlete human evidence, clinical human evidence, and preclinical mechanistic evidence.

Notes: HRV, heart rate variability; zeitgebers, external time cues such as light, meals, and exercise. The checklist synthesizes recurring design issues identified across the chrono-exercise literature, including chronotype and objective phase assessment [3,4,10,42,44,51,52,53,75,76,77,84,85,86,87], sleep/recovery and session placement [11,12,13,14,22,25,35,36,37,38,39,40,41,88,89,90,91,92], mechanistic and sampling considerations [27,54,72,93,105,110,119,120,121], light/nutrition/travel context [17,42,46,47,48,55,78,79,80,81,82,83], warm-up and thermal context [58,59,60], sex-specific context [111,112,113,114,115,116,117], competition-calendar density [44,45,46,47,48], and phase-specific adaptation [125]. Recommended practice refers to the minimum reporting information needed to interpret chrono-exercise studies, not an exhaustive methodological ceiling.

## Data Availability

No new data were created or analyzed in this study. Data sharing is not applicable to this article.

## Abbreviations

AMPK	AMP-activated protein kinase
BMAL1	brain and muscle ARNT-like 1
CLOCK	circadian locomotor output cycles kaput
CRY	cryptochrome
HRV	heart rate variability
HIF-1alpha	hypoxia-inducible factor 1alpha
IJMS	International Journal of Molecular Sciences
MFN2	mitofusin 2
NAD+	nicotinamide adenine dinucleotide
NRF2	nuclear factor erythroid 2-related factor 2
PER	period
PGC-1alpha	peroxisome proliferator-activated receptor gamma
ROS	reactive oxygen species
RPE	rating of perceived exertion
SCN	suprachiasmatic nucleus
SIRT1	sirtuin 1
